# Sequential Outbreaks Due to a New Strain of Neisseria Meningitidis Serogroup C in Northern Nigeria, 2013-14

**DOI:** 10.1371/currents.outbreaks.b50c2aaf1032b3ccade0fca0b63ee518

**Published:** 2014-12-29

**Authors:** Anna Funk, Kennedy Uadiale, Charity Kamau, Dominique A. Caugant, Umar Ango, Jane Greig

**Affiliations:** Médecins sans Frontières, Sokoto, Nigeria; Nigeria Emergency Response Unit (NERU), Médecins sans Frontières, Sokoto, Nigeria; Médecins sans Frontières, Amsterdam, Netherlands; WHO Collaborating Centre for Reference and Research on Meningococci, Norwegian Institute of Public Health, Oslo, Norway; Sokoto State Ministry of Health, Sokoto, Nigeria; Manson Unit, Médecins Sans Frontières, London, United Kingdom

**Keywords:** disease outbreak, infectious disease, meningococcal disease, Neisseria meningitidis, Nigeria

## Abstract

Background Neisseria meningitidis serogroup C (NmC) outbreaks occur infrequently in the African meningitis belt; the most recent report of an outbreak of this serogroup was in Burkina Faso, 1979. Médecins sans Frontières (MSF) has been responding to outbreaks of meningitis in northwest Nigeria since 2007 with no reported cases of serogroup C from 2007-2012. MenAfrivac®, a serogroup A conjugate vaccine, was first used for mass vaccination in northwest Nigeria in late 2012. Reactive vaccination using polysaccharide ACYW135 vaccine was done by MSF in parts of the region in 2008 and 2009; no other vaccination campaigns are known to have occurred in the area during this period. We describe the general characteristics of an outbreak due to a novel strain of NmC in Sokoto State, Nigeria, in 2013, and a smaller outbreak in 2014 in the adjacent state, Kebbi. Methods Information on cases and deaths was collected using a standard line-list during each week of each meningitis outbreak in 2013 and 2014 in northwest Nigeria. Initial serogroup confirmation was by rapid Pastorex agglutination tests. Cerebrospinal fluid (CSF) samples from suspected meningitis patients were sent to the WHO Reference Laboratory in Oslo, where bacterial isolates, serogrouping, antimicrobial sensitivity testing, genotype characterisation and real-time PCR analysis were performed. Results In the most highly affected outbreak areas, all of the 856 and 333 clinically suspected meningitis cases were treated in 2013 and 2014, respectively. Overall attack (AR) and case fatality (CFR) rates were 673/100,000 population and 6.8% in 2013, and 165/100,000 and 10.5% in 2014. Both outbreaks affected small geographical areas of less than 150km2 and populations of less than 210,000, and occurred in neighbouring regions in two adjacent states in the successive years. Initial rapid testing identified NmC as the causative agent. Of the 21 and 17 CSF samples analysed in Oslo, NmC alone was confirmed in 11 and 10 samples in 2013 and 2014, respectively. Samples confirmed as NmC through bacterial culture had sequence type (ST)-10217. Conclusions These are the first recorded outbreaks of NmC in the region since 1979, and the sequence (ST)-10217 has not been identified anywhere else in the world. The outbreaks had similar characteristics to previously recorded NmC outbreaks. Outbreaks of NmC in 2 consecutive years in northern Nigeria indicate a possible emergence of this serogroup. Increased surveillance for multiple serogroups in the region is needed, along with consideration of vaccination with conjugate vaccines rather than for NmA alone.

## Background

In the African meningitis belt, and specifically within northern Nigeria, most meningitis outbreaks have been caused by *N. meningitides* serogroup A (NmA), including large (10-100 thousand cases) and widespread outbreaks 1 ^,^ 2 ^,^ 3 ^,^ 4. In the past 15 years, there have been an increasing number of large outbreaks caused by *N. meningitidis* serogroups W135 and X 5 ^,^ 6 ^,^ 7 ^,^ 8 ^,^ 9. Outbreaks due to *Neisseria meningitidis* serogroup C (NmC) have also occurred but were smaller and less frequent than NmA outbreaks 4. The last NmC outbreak in this region occurred in 1979 in Burkina Faso with 539 cases reported (attack rate (AR) 517/100,000) 10. Outbreaks caused by NmC in northern Nigeria are rare, with the last and only recorded outbreak in 1975 with no detailed report published 4. Other notable NmC outbreaks occurred in the 1970s in Sao Paulo, Brazil and Ho Chi Minh, Vietnam with 2005 (11/100,000 people) and 1015 (>20/100,000 people) cases respectively 4. In the USA, morbidity and mortality are higher among young adults in outbreaks caused by NmC compared with other serogroups 11.

Médecins sans Frontières (MSF) has conducted surveillance and response to cerebrospinal meningitis (CSM) outbreaks in northwest Nigeria since 2007. Meningitis outbreaks due to NmA in northwest Nigeria in 2008 and 2009 were recorded with 7601 and 9442 cases, respectively; MSF carried out reactive vaccination using polysaccharide ACYW135 vaccine in affected parts of the region in these years. An outbreak due to serogroup W135 occurred in 2010 with 2307 cases. From 2007-2012, MSF recorded no outbreak caused by NmC in the region. In December 2012, the most recent mass vaccination for meningitis in northwest Nigeria, conducted by the National Primary Health Care Development Agency (NPHCDA) of the Ministry of Health (MoH), the World Health Organization (WHO) and donor organizations, used MenAfrivac®, a serogroup A conjugate vaccine 12.To our knowledge, there has been no mass vaccination specifically targeting NmC alone in this region.

This paper describes the general characteristics of an outbreak due to a novel strain of NmC in Sokoto State, Nigeria in early 2013 and a smaller outbreak of the same strain in 2014 in the adjacent state, Kebbi, during which time no other serogroups were confirmed in the region.

## Methods

**Case definition**. During this outbreak, the case definition used for CSM for those over 1 year of age was sudden onset fever and either neck stiffness or petechial rash. For infants under 1 year of age the case definition was sudden onset fever and either bulging fontanelle or petechial rash. Only cases adhering to this case definition were treated, had CSF samples taken, and were recorded as a suspected meningitis case.

**Data collection**. In the four northwestern states of Nigeria, meningitis surveillance is done by the MSF Surveillance Nurse through weekly proactive contact with all government disease notification officers. There is one notification officer for each Local Government Area in each state, and they are required to contact all health posts in their jurisdiction each week. The MSF Surveillance Nurse relays reports of meningitis cases to the MSF Emergency Response Unit for follow-up and confirmation using clinical and laboratory criteria. At MSF meningitis case-management sites, information for each case was recorded in a standardized line-list of core data. Maps of the outbreak area were created using data from case tracing. Affected population estimates were derived by combining the known population, as per the most recent national census, for each ward which had at least one case.

The aggregated data used for this paper were collected as part of routine activities which MSF has approval to conduct from the MOH. This work met the standards set by the independent MSF Ethics Review Board for retrospective analyses of routinely collected programmatic data 13.

**Laboratory methods**. ****Cerebral spinal fluid (CSF) samples were collected from all eligible suspected cases at the start of the outbreak and tested using the rapid Pastorex® latex agglutination kit. Pastorex test kits were kept in controlled, refrigerated storage, between 2 and 8 degrees Celsius. Cold chain procedures were maintained while transporting test kits to the field, in Gio’Style boxes with ice-packs. The field team conducted quality control tests on the kits with each usage, and returned the kits to refrigerated storage at the end of each day. The first 21 and 17 samples in 2013 and 2014, respectively, were inoculated into Trans-isolate media 14 and sent to the WHO Collaborating Centre for Reference and Research on Meningococci, Oslo, for confirmation. Bacterial identification was determinedby Gram staining, the oxidase reaction and standard biochemical tests. The strains were stored at –80°C in brain heart broth with 15% sterile glycerol or in Greaves solution. *N. meningitidis* strains were serogrouped by slide agglutination with commercial antisera (Remel, GA, USA) 15. Antimicrobial susceptibility testing was performed by determination of the minimal inhibitory concentrations (MIC) using Etest (AB Biodisk, Solna, Sweden). Isolates were tested for susceptibility to penicillin G, amoxicillin, ceftriaxone, ciprofloxacin, chloramphenicol, rifampin, tetracycline and sulphonamides, and classified using the breakpoints from the European Committee on Antimicrobial Susceptibility Testing 16.

***Genotypic characterization:*** DNA from each strain was prepared by suspending bacteria in Tris-EDTA buffer (10 mM Tris-HCl and 1 mM EDTA), pH 8.0, heating at 95°C for 10 min, and followed by centrifugation at 16,000 x *g* for 5 min. The supernatant was used as DNA template for PCR. Multi-locus sequence typing (MLST) was performed as described on the MLST website 17. The DNA sequences were compared with those on the MLST website for determination of the allele numbers, STs, and clonal complexes of the isolates 18. Variation in the *porA* and *fetA* genes, coding for the outer membrane proteins PorA and FetA, respectively, was determined by DNA sequencing, as described previously 19 ^,^ 20 . New MLST alleles and STs were submitted to the MLST database 17 together with the strain serogroup and *porA* and *fetA* sequences. PCR analysis of the genes coding for the polysaccharide capsule was performed for genogroup determination of non-serogroupable isolates as described 21.

***PCR analysis of culture negative specimens:*** DNA from Trans-isolate supernatants was purified using QiAmp DNA mini kit (Qiagen) and analysed by real-time PCR for species identification, followed by genogrouping if *N. meningitidis* was identified. Determination of the PorA variant was done by DNA sequencing of the *porA* gene using a nested *porA*-PCR 22.

**Data analysis**. Line-lists were entered into an MSF standardized database in Microsoft Excel. Quality checks on the data were done weekly. Epidemiological curves and frequency summaries of patient history and symptoms were generated in Excel.

## Results

**Case Numbers and Attack Rates, 2013.** During the 20 weeks from February 9^th^ until June 23^rd^, 2013, a total of 856 suspected cases of CSM presented for treatment at MSF or MoH treatment sites in Sokoto State (Table 1). The attack rate was 673 cases per 100,000 population in the affected wards of the state (Figure 1). Fifty-eight (58) deaths were recorded from treatment centres, giving a case fatality rate (CFR) of 6.8%. During the same period in 2013, some CSM cases were reported and treated by the MoH in Kebbi State, which borders Sokoto to the West. Detailed information on the cases from Kebbi State is not available.

**Table 1: All suspected clinical cases and death numbers from the most affected state, with attack rates and case fatality rates for each year.** †Population figures are a combination of the population, as per most recent census, of all affected wards which had at least one case.

Year	State most affected	Total population Affected†	Total number of cases	Attack Rate (per 100,000)	Total number of deaths	Case fatality rate (%)
2013	Sokoto	127,097	856	673	58	6.8
2014	Kebbi	201,457	333	165	35	10.5

**CSM cases presenting for treatment at MSF supported treatment centres in Sokoto state, 2013, by week of presentation.**
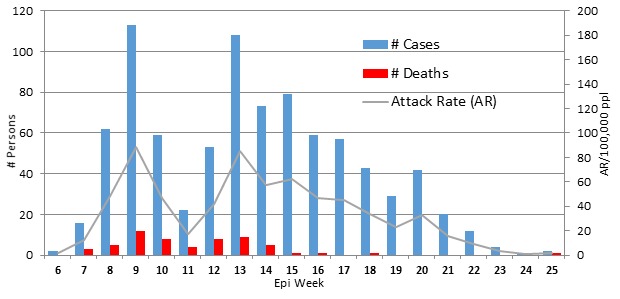

**Case Numbers and Attack Rates, 2014.** In 2014, over the 8 weeks from March 14^th^ until May3^rd,^ 333 cases of suspected CSM from Kebbi State presented for treatment at MSF-supported sites. The overall attack rate during this outbreak was 165 cases per 100,000 population in the affected wards. The CFR was 10.5% (35 deaths). One suspected case also presented at a treatment site in Kebbi from the adjacent Sokoto State during this time.

**Outbreak Demographics and Spread, 2013-2014. **Of 856 cases in 2013, age and sex information were available for 826 (96%);** 404 (49%) were female and 422 (51%) male. The highest proportions of cases were found in those aged 5-14 years (n=361; 44%) and 15-29 years (n=235; 29%). In 2014, the sex distribution was similar (53% male) to that in 2013, and similarly, those aged 5-29 years constituted about three-quarters of cases (57% [5-14 years]; 20% [15-29 years]). The 2013 outbreak was limited to a small geographical area, spreading gradually to 44 villages and remaining restricted to a region of 105km^2^ (Figure 2). The epidemic curve for the 2013 outbreak (Figure 1) shows a peak in the 9th epidemiological week, during which time the outbreak was mostly restricted to the two index villages with high attack rates. Case numbers in these villages then decreased, leading to the low attack rate seen in the 11th week. The increase in weeks 12 and 13 reflects presentations from areas outside the index villages. The gradual overall decline in cases after this period seems to be due to the subsequent serial rise and fall of cases in other villages. The 2014 outbreak was slightly more widespread, affecting 57 villages in a region of approximately 150km^2^.

**Affected villages and outbreak spread in Sokoto State in 2013:**
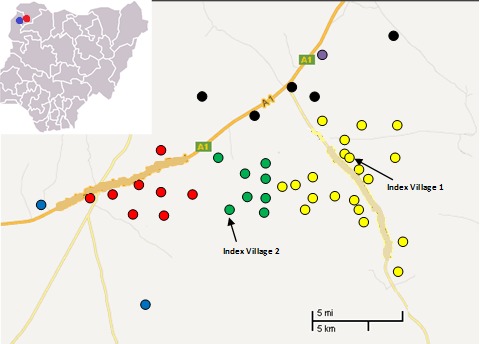
Each coloured dot represents a village which experienced at least one case in 2013, colour differs based on administrative ward. General location of outbreaks in Northwest Nigeria is indicated with a red dot (2013 outbreak) and a blue dot (2014 outbreak) on the full country map at top left.

**Laboratory Results, 2013-2014.** In the 2013 outbreak, out of the 856 clinically suspected cases, MSF tested 77 CSF samples with rapid Pastorex latex agglutination tests; 27 were positive for NmC, and 50 were negative. In 2014, out of the 333 clinically suspected cases, MSF tested 27 CSF samples by Pastorex test; 13 were positive for NmC and 14 were negative. 21 and 17 Trans-Isolate media inoculated with CSF from suspected meningitis patients were sent to the reference laboratory in Oslo in 2013 and 2014, respectively (Table 2). In total, 11 (52%) and 10 (59%) samples were confirmed as serogroup C in 2013 and 2014, respectively. Most were also confirmed as a new strain with sequencing of ST-10217 PorA type P1.21-15,16 and FetA type F1-7. No other serogroups were identified during testing of CSF samples from these outbreaks.

**Table 2: Analyses of the CSF samples sent in Trans-Isolate medium to the Collaborating Centre for Reference and Research on Meningococci, Oslo:** RT-PCR = Real-Time Polymerase Chain Reaction †For two samples the serogroup was not confirmed by RT-PCR due to low bacterial load, but the PorA type suggests that the same strain was involved.

Year	Number of CSF samples	Number of samples positive for NmC in culture	PorA type	FetA type	Sequence type	Number culture-negative samples positive for NmC in RT-PCR	PorA type of RT-PCR positive samples
2013	21	7	21-15,16	F1-7	10217	6†	All 6 samples: 21-15,16
2014	17	5	21-15,16	F1-7	10217	5	4 samples: 21-15,16 1 sample: 21-15,16-50

## Discussion

These outbreaks were caused by a strain of NmC that has not been seen anywhere else in the world: sequence ST-10217 PorA type P1.21-15,16 and FetA type F1-7. As far as we know this is the first meningitis outbreak caused by NmC in northern Nigeria since 1975 and in the meningitis belt since 1979 4 ^,^ 10 ^,^ 23 . The seasonal pattern and presentation of these outbreaks, in the dry period during and following the Harmattan winds, did not differ much from those of other meningitis outbreaks caused by *N. meningitidis* in sub-Saharan Africa 24 ^,^ 25 ^,^ 26 The outbreaks were confined to relatively small areas, and did not have the ‘wild fire’ effect more typical of meningitis outbreaks caused by NmA 2 ^,^ 4. Age groups with the highest proportion of cases in these outbreaks were 5-14 years and 15-29 years, also typical of meningitis, with these ages possibly exposed to more risk factors for transmission such as overcrowding and active and passive smoke exposure 11 ^,^ 27 ^,^ 28 .

A relatively high percentage of Pastorex latex agglutination tests, carried out only on patients fitting the clinical case definition, had negative results during both the 2013 (64%) and 2014 (48%) outbreaks. This may have occurred for a number of reasons, including meningitis symptoms with non-bacterial cause, or self-medication with antibiotics or traditional medicines prior to presentation. As there is no other report of Pastorex testing being used in a real outbreak situation for NmC, it is uncertain whether these negative test result percentages are unusually high.

During the 2013 outbreak, the MSF control strategy consisted of active case finding and health promotion activities. Due to heightened security issues and anti-vaccination sentiments in the area, reactive vaccination was not done. It is not clear that health promotion activities significantly decreased outbreak spread, though it was observed that after initiation of this strategy, cases presented earlier to treatment centres and this could have contributed to lower mortality rates in the latter part of the outbreak. In 2014, the Kebbi state MoH, along with the WHO, attempted twice to apply for vaccines from the International Coordinating Group; however, neither request was granted. The state MoH later received approximately 20,000 doses of ACYW135 polysaccharide vaccine from the Federal MoH, which was used for reactive vaccination in some affected villages; details of vaccination strategy and outcomes were not available to MSF. Similar to the previous year, health promotion and active case finding was carried out by MSF teams during the 2014 outbreak. Control strategies employed in 2014 by MSF and the MoH did not clearly impact outbreak spread.

The 2014 NmC outbreak had fewer cases than the 2013 outbreak; however, since the outbreaks occurred in different regions and were controlled with different measures we cannot say this signifies a pattern of decrease. It is possible that these small, localized outbreaks will precede increasingly widespread occurrence of meningitis due to this serogroup (C) in the meningitis belt, as has been noted as a characteristic *N. meningitidis* outbreak pattern^4^ .

It is possible that the mass vaccination with a conjugate ‘A’ vaccine (MenAfriVac®) in this region a few months prior to the 2013 outbreak could have had an influence on the emergence of new strains or less commonly seen serogroups, such as NmC. Serogroup replacement following mass meningitis vaccination has been noted in west Africa; reports from Niger and Burkina Faso have indicated a significant increase in serogroup W prevalence in the years following campaigns with MenAfriVac® around 2010^29^^,^ 30. Following a mass vaccination with MenAfriVac® in Chad in 2011/2012 it was seen that in one community serogroup A carriage decreased from 0.7 to 0.02%, while carriage of “other” serogroups (ie. not A, W, X) increased from 0.4 to 0.7%**31**. Because of the possibility of serogroup replacement following vaccination, enhanced surveillance systems in the region are a priority^32^ ****.

## Conclusions and Recommendations

NmC outbreaks have emerged in northwest Nigeria in the past 2 years, and there is some evidence of serogroup replacement in the meningitis belt following recent mass vaccination with NmA conjugate vaccine. Meningitis case surveillance systems, for both serogroup and strain, should continue to be strengthened in this region to allow for early identification and proper control (such as vaccination for the appropriate serogroup) of outbreaks. If NmC outbreaks become more widespread in northern Nigeria or adjacent regions in the coming years, large-scale preventative action may be required; a key measure is to ensure availability of ACYW135 polysaccharide vaccine for reactive vaccination.

## Competing Interests

The authors have declared that no competing interests exist.
